# Management of retained endoscopy capsule: a case series and literature review

**DOI:** 10.1093/jscr/rjae749

**Published:** 2024-11-25

**Authors:** Preston H Palm, Madison M Patrick, Claudia A Cruz, Udayakumar Navaneethan, Antonio Caycedo, Marco Ferrara

**Affiliations:** Orlando Health Colon and Rectal Institute, 110 W. Underwood St, Ste A, Orlando, FL 32806, United States; Department of Clinical Sciences, Florida State University College of Medicine, 250 E. Colonial Dr. #200, Orlando, FL 32801, United States; Department of Clinical Sciences, Florida State University College of Medicine, 250 E. Colonial Dr. #200, Orlando, FL 32801, United States; Orlando Health Colon and Rectal Institute, 110 W. Underwood St, Ste A, Orlando, FL 32806, United States; Orlando Health Colon and Rectal Institute, 110 W. Underwood St, Ste A, Orlando, FL 32806, United States; Orlando Health Colon and Rectal Institute, 110 W. Underwood St, Ste A, Orlando, FL 32806, United States

**Keywords:** retained capsule endoscope, video capsule endoscopy, Crohn’s disease, intestinal obstruction, endoscopy, enteroscopy

## Abstract

Video capsule endoscopy has become the gold standard for the evaluation of small bowel pathology. Capsular retention remains the most significant risk of this intervention. Here, we present two cases of retained capsules and our minimally invasive approach to retrieval. We also review the literature pertaining to retained endoscopy capsules and highlight a range of medical, surgical, and preventative strategies utilized in its management.

## Introduction

Since its introduction by Iddan *et al*. in Nature and approval by the FDA in 2000 [1, 2], video capsule endoscopy (VCE) has evolved to become the gold standard for evaluating small bowel diseases [3]. While investigating intestinal bleeding is its most common use case, its indications have expanded to include the investigation of Crohn's disease (CD), iron deficiency anemia, malabsorptive syndromes, intestinal lesions related to nonsteroidal anti-inflammatory (NSAID) use, small intestinal tumors, and the surveillance of polyposis syndromes [2, 4–7]. It is generally contraindicated in known or suspected gastrointestinal (GI) obstruction, stricture, fistula, swallowing disorders, implanted electronic medical devices, and pregnancy [2].

The main risk associated with the implementation of VCE is capsule retention (CR). This is defined as the presence of the capsule endoscope in the digestive tract for a minimum of 2 weeks, requiring targeted medical or surgical intervention for removal [8]. The majority of studies have reported a frequency of CR between 0.3% and 2.5% across a range of indications [9, 10]. Patients presenting with symptoms of small bowel obstruction have been shown to be at the highest risk of CR, with a documented incidence of 21% [11]. Those with known CD are at high risk of CR as well with reported rates between 2.6% and 13% [7, 11]. Patients with suspected CD have demonstrated a risk of CR between 0.5% and 6.3% [4, 12]. Those with definite or suspected small bowel ulcers, neoplastic lesions, enteropathy, dysmotility disorders, stenosis, a history of abdominal surgery, adhesions, or heavy NSAID use have also been reported as being at elevated risk for CR [13, 14]. Patients in which VCE was utilized for the indication of obscure GI bleeding actually had a lower risk of CR with a rate of 1.2%–1.5% [3, 4, 7].

The most common site of retention is the small bowel—primarily the ileum—followed by the esophagus [3, 4, 15]. The majority of CR patients are asymptomatic, with most studies finding 0%–38% of patients feeling symptoms of obstruction due to their retained capsule [3–5, 16]. However, two studies did report higher rates of 59% and 75% [9, 17]. Prolonged CR has been associated with obstruction, perforation, and scarring related to fragmentation of the capsule resulting in the need for intervention [2]. Here, we describe two cases of patients with CR and their subsequent management.

## Case series

**Figure 1:**
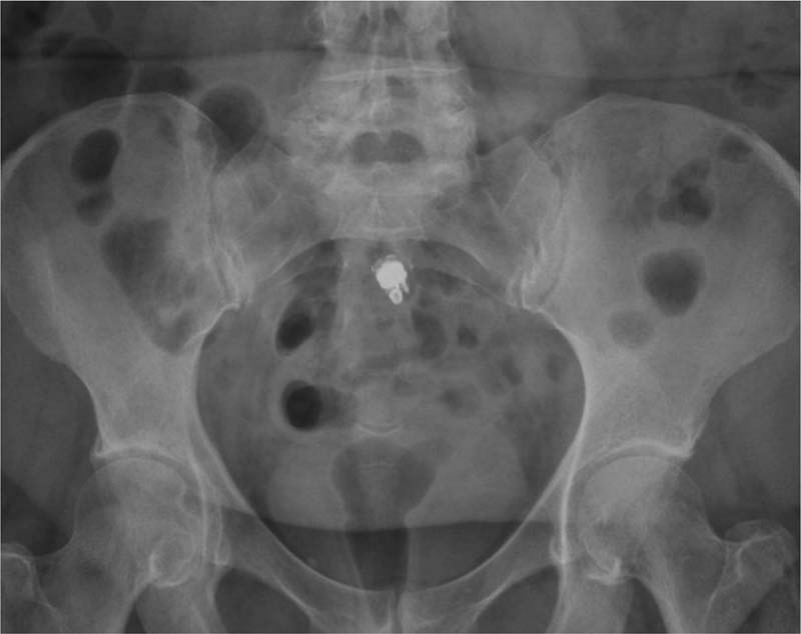
Radiographic appearance of retained capsule in terminal ileum.

A 73-year-old male with a history of hypertension and CD presented to an outside facility in September 2022 with an ileal stricture and microperforation in the setting of ileitis. The patient underwent exploratory laparotomy for resection of the involved segment with reanastomosis. Pathology was positive for adenocarcinoma in a field of high-grade dysplasia and inflammatory changes consistent with Crohn’s. Two months later, the patient underwent VCE with an outside gastroenterologist. The capsule was retained at the distal ileum. Two weeks later, a colonoscopy revealed a stricture in the terminal ileum that could not be traversed, and the capsule was not able to be visualized. A CT scan showed the capsule lodged proximal to his anastomosis near the ileocecal valve. Despite a 25-day steroid taper, the capsule remained static on imaging over the next 3 months (Fig. 1). During that interval, the patient remained asymptomatic. He was eventually referred to our clinic for surgical retrieval. After a discussion of the risks, benefits, and alternatives of surgery, the patient consented to a robotic ileocolectomy for retrieval of the capsule.

**Figure 2:**
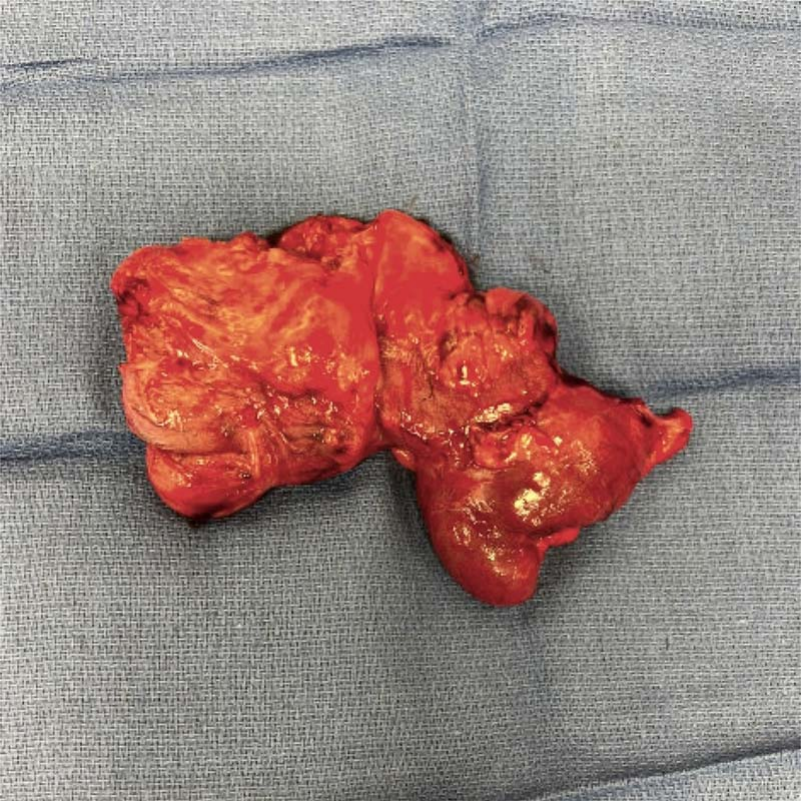
Resected bowel segment.

**Figure 3:**
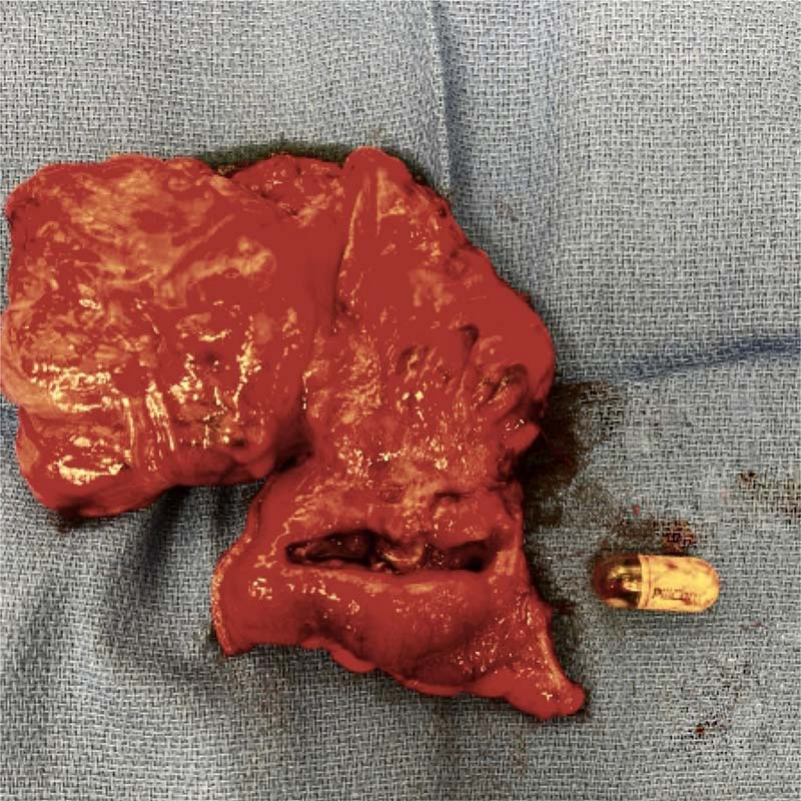
Retained capsule.

In the operating room, the patient was positioned in the lithotomy position. The abdominal cavity was entered with an optical trochar at Palmer’s point. Additional trocars were placed in the left side of the abdomen, a bilateral transversus abdominis plane (TAP) block was performed, and the robot was docked. After the lysis of adhesions, the location of the capsule was confirmed proximal to his recent anastomosis via direct visualization. A robotic blue load stapler was used to divide the ileum proximal to the capsule. The mesentery was taken down up onto the ascending colon using a robotic vessel sealer. The proximal ascending colon was divided with another blue load stapler. IndoCyanin green (ICG) and Firefly™ were employed to confirm adequate blood supply to the distal ileum and ascending colon and an isoperistaltic stapled anastomosis was created. The specimen was extracted via a Pfannenstiel incision. The specimen was incised on the back table (Fig. 2), and the capsule was removed intact (Fig. 3). Post-operatively, the patient progressed without complications and was discharged home on post-operative Day 2. He has subsequently been seen in follow-up and continues to do well.

Our second case involved a 72-year-old female undergoing a workup for chronic anemia at an outside facility. After negative esophagogastroduodenoscopy and colonoscopy in February of 2023, her gastroenterologist performed a VCE. This initially revealed multiple mucosal ulcerations and strictures within the small bowel but subsequently became retained in the ileum as confirmed on CT. The patient was initially referred to an advanced GI endoscopic interventionist. Double balloon enteroscopy was performed, but the capsule was unable to be reached with this approach. The patient was referred to our colorectal surgery group for definitive management. At the time of her pre-operative visit—~1 month from her VCE procedure—the patient remained asymptomatic. After a discussion of the risks, benefits, and alternatives of surgery, the patient consented to a laparoscopic small bowel resection with planned retrieval of the capsule.

**Figure 4:**
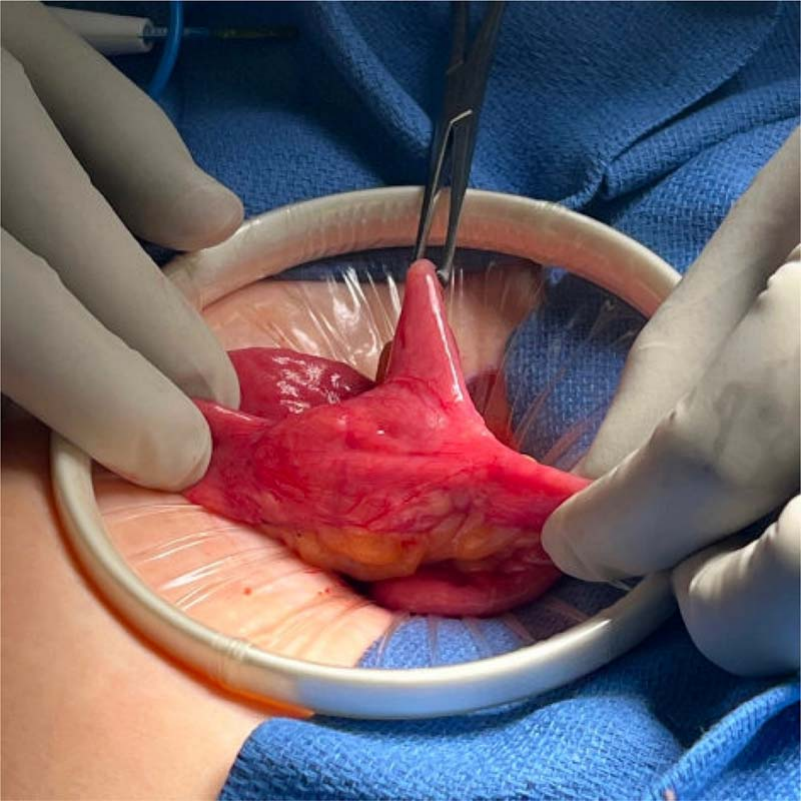
Meckel’s diverticulum.

**Figure 5:**
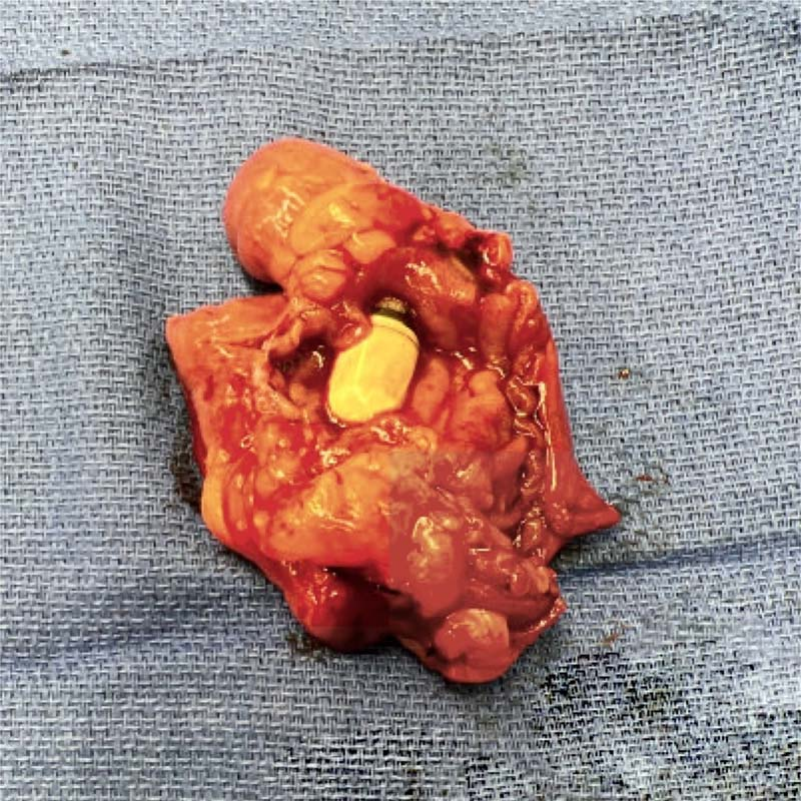
Capsule and Meckel’s.

In the operating room, the patient was placed in lithotomy position. The abdomen was entered via Palmer’s point with an optical trochar. A bilateral TAP block was performed. Additional trocars were placed along the left side of the abdomen, and the patient was positioned in Trendelenburg. After running the bowel, a Meckel’s diverticulum was identified 40 cm from the ileocecal valve with surrounding small bowel thickening and inflammation. Evidence of the retained capsule was identified immediately proximal to the Meckel's diverticulum. The bowel was extracorporealized through a Pfannenstiel incision, and the capsule position was confirmed with palpation (Fig. 4). A blue load GIA 75 stapler was used to divide the bowel proximal and distal to the Meckel's diverticulum. The intervening mesentery was taken using clamps and silk ties, and a stapled antiperistaltic anastomosis was created. The specimen (Fig. 5) was sent to pathology and the Pfannenstiel was closed. Post-operatively, the patient progressed appropriately and was discharged home on post-operative Day 1. Her pathology confirmed a Meckel’s diverticulum with a chronic appearing ulcer adjacent to the diverticulum. She has subsequently been seen in follow-up and continues to do well.

## Discussion

Capsule endoscopy has become the gold standard for evaluating small bowel diseases. Its use has expanded since its introduction in 2000 to encompass a wide range of indications including workup of GI bleeding and CD [3, 7]. CR remains a persistently recognized risk of this procedure. This case series conveys a single center’s approach to managing this known complication. However, a range of approaches have been described.

Conservative approaches to the management of CR have been utilized with varying levels of success. One approach is observation alone. In a multicenter retrospective review by Fernández-Urien *et al*. [3] of 5428 VCE procedures, 104 procedures (1.9%) were complicated by CR. Thirty-nine of these capsules (37.5%) passed without procedural intervention at a mean of 42 days. In another multicenter retrospective review of 2921VCE procedures, 61 procedures (2.1%) were complicated by CR. [18] Thirty-two of these (52%) were safely observed and eventually passed without procedural intervention. In a third multicenter retrospective review of 2705 VCE procedures, 20 (0.7%) were complicated by CR [15]. Three of these (15%) were safely observed and eventually passed without procedural or medical intervention at a mean of 59 days. Medical intervention has been demonstrated to play a role as well, with 2 of the above 20 cases (10%) of CR resolving with steroid treatment alone. Similarly, Fernández-Urien *et al*. [3] found that they were able to successfully manage 16% (17/104) of the cases of CR with steroids alone. Three of 104 (3%) cases of CR were managed successfully with laxatives alone.

Endoscopic management has been utilized extensively for the retrieval of retained VCE. Double balloon enteroscopy consists of a long endoscope and a flexible overtube with latex balloons attached at the tip of the endoscope and the overtube. By utilizing a push-and-pull technique to place the small bowel segments onto the overtube step-by-step, operators may explore the small bowel and retrieve foreign bodies such as retained endoscopy capsules [19]. Double balloon enteroscopy has proven to be a safe and reliable technique for the retrieval of retained capsules [20]. It has significantly reduced the need for operative interventions in patients with CR. Nevertheless, there remains a subset of capsules that are unable to be retrieved in this manner.

Surgical management continues to play an important role in the retrieval of retained capsules. Surgical intervention is an obvious choice for patients who are acutely symptomatic such as those with obstructions or bleeding related to their retained capsule. However, it may also be used to prevent such complications where medical and endoscopic approaches fail. Laparotomy for retrieval of retained capsules was described as early as 2005 [21]. Since that time, newer, more minimally invasive approaches have been developed to reduce the morbidity of surgical retrieval. Dominguez *et al.* [22] first described a laparoscopic approach for the retrieval of a retained video capsule endoscope in 2006. Since that time, a variety of laparoscopic approaches have been described including single-site and fluoroscopically guided approaches. To the author’s knowledge, this is the first published description of a robotic surgical retrieval technique for retained endoscopy capsules.

Preventative methods exist that may be employed to reduce the risk of capsular retention. Unfortunately, screening candidates for VCE with CT imaging, small bowel follow-through (SBFT), or magnetic resonance enterography (MRE) alone has been demonstrated to be ineffective in reducing the rate of VCE complications [3, 4, 14]. However, radiologic testing has been found to be helpful when used in conjunction with a patency capsule (PC). PCs are dissolvable capsules simulating the dimensions of the actual device that are able to be detected by some external means—such as an radio-frequency idenfication (RFID) scanner or imaging [23]. These were developed to allow physicians to perform CE with greater confidence that the capsule will be safely excreted in patients at risk for CR. Radiologic testing may minimize PC study false-positive results by confirming or excluding the presence of a stricture suspected by the PC and even localizing the PC if it fails to pass in a timely manner [24]. PC use on its own has been demonstrated to be effective at reducing CR rates in patients with known strictures. In a study performed by Herrerias *et al*., a total of 106 patients with known strictures ingested the PC. Fifty-nine (56%) excreted the PC intact and those subsequently underwent CE [23]. There were no cases of CR in this cohort. The American Gastroenterological Association now recommends the use of PC in patients with known or suspected small bowel strictures to minimize the risk of retention [25].

VCE has become the gold standard for investigating small bowel diseases and has a broad range of uses. The primary risk associated with VCE is capsular retention. The risk of CR may be reduced through the implementation of PCs in select populations. This risk may be further reduced with concomitant pre-procedural imaging. Despite these measures, CR remains a potential complication of VCE. CR may be managed with observation, medical intervention, endoscopic procedures, and surgery. Here, we have described a series of cases safely managed with minimally invasive surgical approaches for retrieval of a retained endoscopy capsule.
